# 

**Published:** 1869-11

**Authors:** 


### CONTENTS.

### ORIGINAL COMMUNICATIONS.

Introductory Address to the 24th Annual Session of the Ohio College of Dental Surgery... 449
Odds and Ends... 459
Os-Artificial Impugned... 461
Review... 463

### PROCEEDINGS OF SOCIETIES.

East Tennessee Dental Association... 467
Aluminum... 468
Gun-Cotton... 468

### SELECTIONS.

Influence of Education... 469
Nursing Sore Mouth... 470
Threatened Death from Chloroform... 471
Chleral—A New Anæsthetic... 472
Carbolic Acid as an Antiseptic... 473
The Author of Thesaurus... 473
Advertisements of Specialists... 474
A New Silver Alloy... 474
The Food of a Human Being... 475
Tooth-Brushes... 475
Comparative Mortality of Five Large Cities for September... 476
A Model Document... 476
The Earliest Writer on Carbolic Acid... 476
Weights and Measures... 477
An Improved Compound for Welding... 477
A New Styptic Collodion... 477
Library of the American Medical Association... 478
Prof. Boehm—Dissection Wound... 478
A Definition... 478
Hypodermic Injections... 478
Fracture of the Superior Maxillary Bone... 479
A Worthy Foundation... 481
A Collection of Pins... 481
Phosphoric Acid in Organic Nature... 482
Keenness of the Senses... 482
Scarifying the Gums... 483
Bromide of Potassium Pushed to its Full Extent... 483
The Practice of Medicine in Oregon... 484

### CORRESPONDENCE.

Knoxville, Tenn... 485
To Whom it May Concern... 487

### EDITORIAL.

Bibliographical... 488
Library of Ohio College of Dental Surgery... 489
Vice Versa... 489
Educational... 489
Progress... 490
Dental Luminary... 490
The New Masterpiece... 491
Ohio State Dental Society... 491
To the Members of the American Dental Association... 492

